# Fenugreek and Its Effects on Muscle Performance: A Systematic Review

**DOI:** 10.3390/jpm13030427

**Published:** 2023-02-27

**Authors:** Waleed I. Albaker

**Affiliations:** Department of Internal Medicine and Endocrinology, College of Medicine, Imam Abdulrahman Bin Faisal University, Alkhobar 34224, Saudi Arabia; wialbakr@iau.edu.sa

**Keywords:** muscle, recovery, exercise, athletes, fenugreek

## Abstract

Fenugreek extracts possess promising physiological and pharmacological properties in human and animal models. This review aims to provide a scientific and comprehensive analysis of the literature on the effects of fenugreek extracts on muscle performance. An extensive online search was conducted according to the Preferred Reporting Items for Systematic Reviews and Meta-Analyses (PRISMA) statement guidelines. The main medical and scientific engines were searched for articles from May 1981 to May 2021 to capture all scientific studies focused on the effect of fenugreek on muscle and exercise or sport. Out of 81 studies acquired, six eligible randomized controlled trials (RCTs) were included in the qualitative analysis. Four RCTs observed that fenugreek supplementation had significantly improved muscle strength, repetitions to failure (muscle endurance), submaximal performance index, lean body mass, and reduced body fat. Among the remaining two trials, one reported the significant effect of fenugreek extracts on the rate of muscle glycogen resynthesis during post-exercise recovery; however, the other failed to do so. Those two trials were weak, with a minimal sample size (<10). Further, fenugreek glycoside supplementation with sapogenins and saponins reported substantial anabolic and androgenic activity, influencing testosterone levels and muscle performance. It was useful during eight weeks of resistance training without any clinical side effects. Fenugreek with creatine supplementation improved creatine uptake without the necessity of high carbohydrate intake. Hence, fenugreek extracts can be a helpful natural supplement and ergogenic aid for athletes. However, it is better to be aware of doping and liver and kidney damage before using the fenugreek supplement.

## 1. Introduction

Fenugreek (*Trigonella foenum-graecum Linn*) is a legume belonging to the Fabaceae family. Nearly 175 compounds have been identified in fenugreek seeds [1]. Fenugreek contains active constituents such as steroid saponin compounds, fibers, phenolic acid compounds, protodioscin, flavonoids, hydrocarbons, alkaloids, terpenes, fatty acids glycosides, carbohydrates, amino acids, and their derivatives [2].

**Figure 1:**
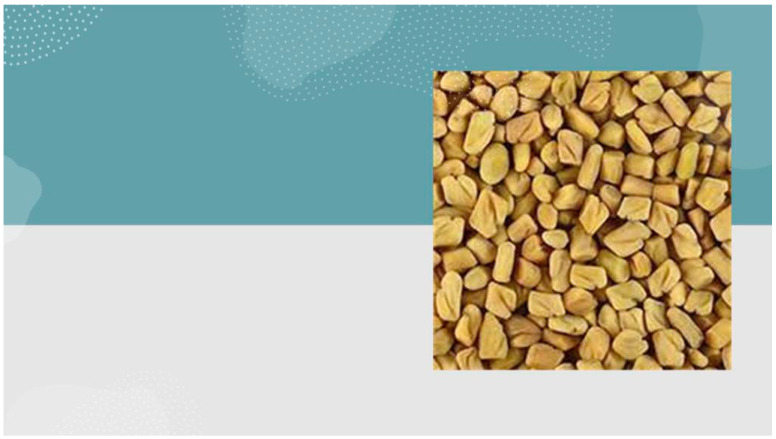
Macroscopic picture of the fenugreek.

**Figure 2:**
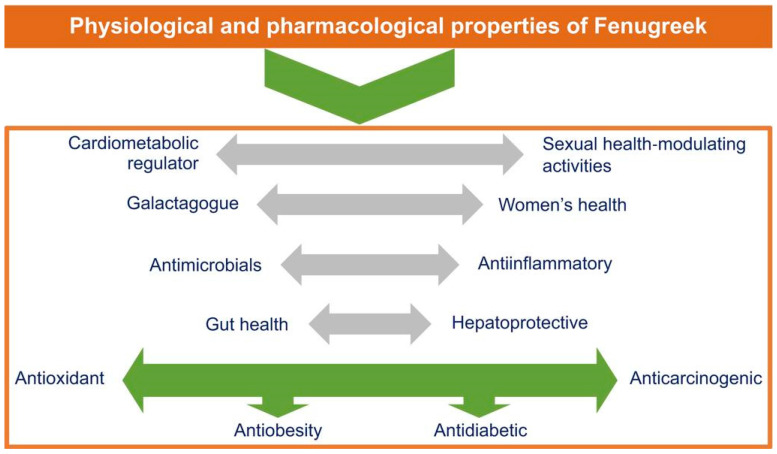
Physiological and pharmacological features of fenugreek.

The genus Trigonella, “little triangle” in Latin, is named after the triangular flowers [3]. It is among the most promising traditional plants, cultivated widely in many parts of Africa, Europe, and Asia as an indigenous medicine, spice, and herbal food [2]. The seeds and leaves of fenugreek are formulated in powder and extract forms for medical purposes [4]. The macroscopic picture of fenugreek is provided in Figure 1. The physiological and pharmacological features of fenugreek are described in Figure 2.

A broad spectrum of systematic reviews and scientific data demonstrated that fenugreek and its extracts improve glycemic and lipid profiles [5]. Fenugreek is known to have antioxidant [6], anti-carcinogenic [7], gastroprotective [8], anti-inflammatory [9], antimicrobial [10], immunological [11], antiobesity [12], and hepatoprotective [13] effects, and are beneficial to women’s health [14]. Further, previous studies reported that fenugreek improved cognitive functions and Parkinson’s symptoms and showed antianxiety, antidepressant, and neuroprotective properties [15,16]. A recent study has reported the beneficial effects of fenugreek and its extract on hormonal-related statuses, such as galactagogue in lactating women and male impotence [17].

The evidence showed that proper nutritional consumption is essential for achieving training adaptations, improving exercise performance, and recovering muscle function [18,19]. Specifically, high athletic performance requires well-controlled nutritional composition and timing (before, during, and after an exercise session) to optimize exercise performance and hasten recovery following exercise [19,20]. Many researchers have identified nutritional supplements that enhance muscle performance and facilitate optimal post-exercise recovery. Therefore, this review aims to provide an update on the recent findings of fenugreek’s muscular, metabolic, and exercise-enhancing effects.

## 2. Materials and Methods

### 2.1. Study Design

**Figure 3:**
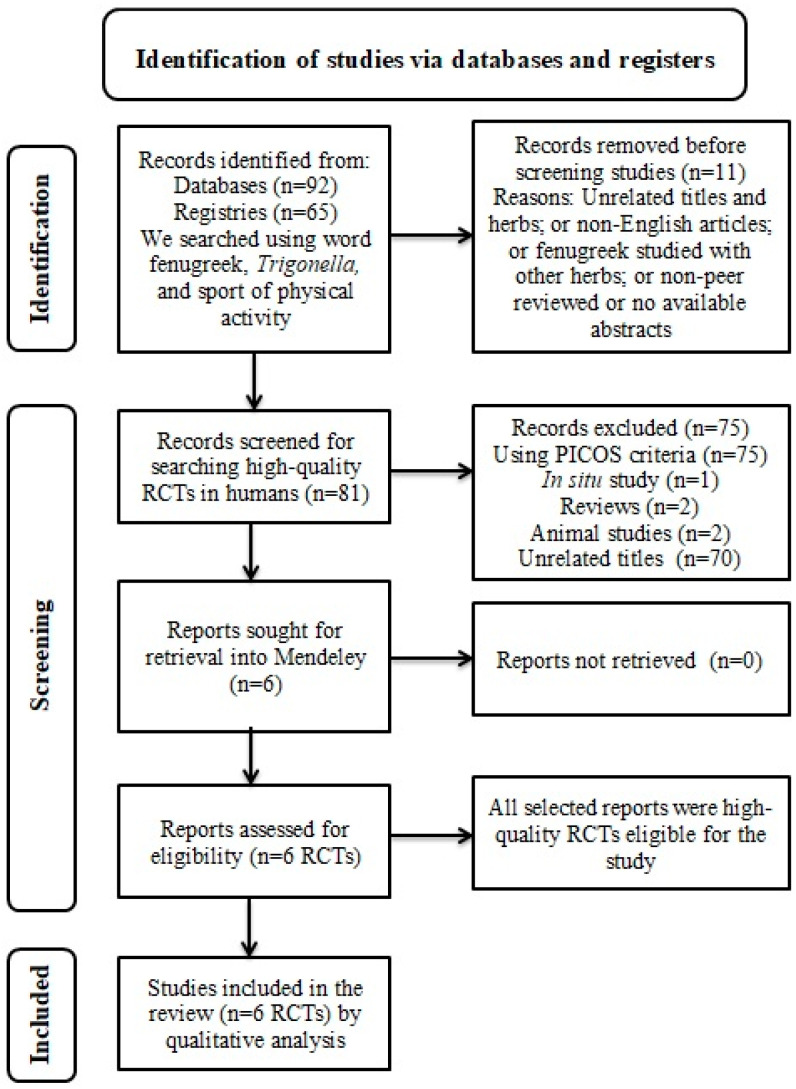
PRISMA flow diagram.

For this review, an extensive online search was conducted according to the guidelines of the Preferred Reporting Items for Systematic Reviews and Meta-Analyses (PRISMA) statement [21]. The PRISMA flow diagram is shown in Figure 3. Further, this systematic review has been registered in the INPLASY, an international platform of registered systematic review and meta-analysis protocols (Registration number: INPLASY202320089). 

### 2.2. Search Strategy

This study searched Google Scholar and MEDLINE/PubMed for articles from May 1981 to May 2021 to capture recent scientific studies about fenugreek and its effects on muscles, exercise, or sport. The keywords used to identify the studies were (((Fenugreek) OR (Trigonella foenum)) AND ((((Sport) OR (Exercise)) OR (Muscle)) OR (Physical activity))) AND ((“1981/05/30”[Date - Publication]: “3000”[Date - Publication])). Further manual screening through Scopus and ISI websites yielded no additional articles. The search was limited to papers written in English. The references retrieved were imported into Mendeley.

### 2.3. Selection Criteria

The systematic review included publications that met the following inclusion criteria: (1) a randomized controlled trial (RCT) with a cross-over or parallel design; (2) assessed the effects of fenugreek on sports, muscle performance, and post-exercise recovery; and (3) reported values at baseline and at the end of follow-up in each group, or the net change data. The exclusion criteria were (1) in vitro, in situ, animal, pregnant women’s, and children’s studies; (2) trials that studied the effect of fenugreek extracts in combination with other herbs; (3) articles that were not peer-reviewed, with no available abstracts, or not written in English. After the preliminary eligibility screening evaluation, only the most complete studies were included.

### 2.4. Data Extraction

A standardized form was used to obtain the following data from each study: (1) general characteristics “first author, publication date, country,” (2) study characteristics “mean age, sample size, sex, study design, daily dose, type of fenugreek/control, duration of the study, conclusion, and limitation” and (3) quality score. As such, the author collected and reviewed the data and recorded it in tabular form.

### 2.5. Quality Assessment

The six-point scale criteria by Hayden et al. [22] was applied to assess the quality of the included studies. The criteria are (1) study participation (the sample is large enough and represents the population of interest); (2) control for confounding factors; (3) determination of the factor of interest (clear definition and description of the factor of interest are provided); (4) study attrition (full explanation of the sample drop out); (5) appropriateness of statistical analysis; and (6) outcome measurement (full explanation of the method used for outcome measurement in such a way that reduces measurement bias). A single-point score was given for each criterion; “the score of 0–3 points indicated low-quality studies, while the score above 3–6 was considered as high-quality studies”.

## 3. Results

Eighty-one studies were obtained from the advanced search. Out of them, 75 were eliminated after thoroughly reviewing the title and abstracts using PICOS criteria. Those excluded studies were (1) in situ study (n = 1); (2) review (n = 2); (3) animal studies (n = 2); (4) combination with other herbs or unrelated titles (n = 70). Finally, six eligible RCTs were included in the qualitative analysis (Figure 3).

### 3.1. Characteristics of the Included Studies

The characteristics of the RCTs are presented in Table 1. Of the six included RCTs, only one trial was conducted in both sexes [23], whereas the remaining RCTs were conducted in males [4,24,25,26,27] from India [27] and the USA [4,23,24,25,26].

### 3.2. Findings

Based on the findings, one trial used fenugreek seed power as an intervention, with daily doses of 500 mg/day for eight weeks (4). Three trials chose fenugreek seed extracts at doses of 2–900 mg/day [24,25,26]. The glycoside fraction of fenugreek seeds was used daily (300 mg) for eight weeks [27]. On the other hand, fenugreek soluble fibers were administered at a daily dosage of 300 mg for 28 days [23].

Four trials reported positive effects of fenugreek on muscle performance and post-exercise recovery [4,23,24,27]. While reviewing those trials, one trial found that fenugreek extract (500mg) had positively affected the upper (1 repetition maximum [RM] bench press) and lower (1 RM leg press) body strength compared to the placebo, without any reported side effects, in eight weeks of resistance training for male participants. It also showed a significant improvement in lean body mass and body fat reduction, thereby augmenting body composition compared to the placebo group [4]. Further, another trial concluded that creatine (3.5 g) plus fenugreek extract (900 mg) for eight weeks, along with a structured resistance training program, could significantly affect muscle strength (1 RM bench press; 1 RM leg press) among resistance-trained males as effectively as creatine (5 g) plus dextrose (70 g). Fenugreek with creatine supplementation might be a new method for improving creatine uptake while eliminating the necessity for high quantities of simple carbohydrates [24].

In contrast, the glycoside fraction of fenugreek (*Trigonella foenum-graecum*) seeds (Fenu-FG) failed to increase muscle strength significantly compared to the placebo group. However, Fenu-FG had more substantial anabolic and androgenic activity and significantly showed more repetitions to failure (muscle endurance) during resistance training than the placebo group. It was beneficial during eight weeks of resistance training, without any clinical side effects [27]. Moreover, an increase in one submaximal fatigue threshold was observed in both the fenugreek soluble fiber (FEN) and the CurQfen (CUR, i.e., curcumin plus fenugreek supplementation) groups, without alterations in maximal endurance performance (VO2 peak). The presence of fenugreek improved the submaximal performance index for both FEN and CUR groups [23].

Additionally, two controversial trials on muscle glycogen resynthesis were reported. However, both trials were weak, with a minimal sample size (<10) [25,26]. Among those trials, one trial inferred that a fenugreek extract supplement with dextrose could promote and accelerate the rate of glycogen resynthesis levels following a glycogen-depleting cycle exercise test in trained male cyclists. Grounded on the similarity in blood glucose and insulin between trials, fenugreek extract supplement with dextrose improves muscle glycogen resynthesis without altering circulating insulin [26]. In contrast, another trial concluded that adding fenugreek to post-exercise feeding does not affect muscle glycogen synthesis or subsequent exercise performance (cycling) among normoglycemic male endurance athletes [25].

## 4. Discussion

### 4.1. Body Composition and Muscle Performance

Testosterone is an essential hormone for muscle homeostasis in both men and women. An earlier study has found elevated body fat mass and decreased protein synthesis, muscle strength, and mass in males with testosterone hormone deficiency [28]. Furthermore, it is an essential hormone for sports performance and athletic success because of its anabolic properties. It causes a positive nitrogen balance by decreasing protein breakdown and increasing protein synthesis, resulting in muscle hypertrophy with improved muscle strength and performance [29]. Additionally, exercise, especially resistance training, directly affects protein synthesis and improves muscle mass and development, resulting in increased basal metabolic rate, exercise performance, and fat oxidation [30]. Notably, fenugreek extracts augmented muscle growth and overall rat weight compared to the control groups [31]. In a human trial, fenugreek extract (500 mg) showed a significant improvement in upper and lower body strength, lean body mass, and body fat reduction, thus enhancing overall body composition over the placebo over an eight-week duration without any side effects. The mechanisms accountable for the observed changes must be clarified due to inadequate research concerning fenugreek’s competence in affecting anaerobic exercise output and hormonal alterations in humans and animals. Moreover, no variations were observed within or between fenugreek extract (500 mg) and the placebo group for any measured serum hormone variables—namely, leptin, estradiol, cortisol, and dihydrotestosterone—excluding free testosterone. Additionally, this fenugreek supplement failed to significantly influence hematological variables (i.e., kidney function or muscle damage markers, liver enzymes, cholesterol, proteins, triglycerides, white and red blood cell count) and muscular endurance on leg and bench press. Further research can be warranted to examine various extractions and doses of fenugreek on trained subjects to conclude whether anabolic hormones can be changed and discover the possibility of additional strength and power output adaptations, which could eventually improve exercise performance [4]. Also, Taylor et al. concluded that fenugreek extract (900 mg) with creatine (3.5 g) during an 8-week resistance program significantly influenced muscle strength and body composition in resistance-trained males as similarly as creatine (5 g) with dextrose (70 g). Both groups significantly increased 1 RM bench press, 1 RM leg press, and lean body mass. Notably, the fenugreek and creatine combination increased 1 RM bench press and lean mass after four weeks of resistance training more than the creatine and dextrose combination. This observation might be due to increased creatine absorption and retention resulting from fenugreek intake, which sets earlier training adaptations. Fenugreek might transport creatine into the skeletal muscle similarly to carbohydrates [24].

Furthermore, a clinical trial found positive effects of fenugreek extract in enhancing body composition, endurance capacity, muscle strength, testosterone levels, and mass (hypertrophy) from baseline when used with an eight-week calisthenic regimen for males [32]. An earlier study confirmed that a six-week dietary supplement comprising chitosan, fenugreek, glucomannan, vitamin C, and gymnema sylvestre significantly reduced body weight and fat in obese adults [33]. Fenu-FG supplementation significantly decreased body fat and thigh and triceps skinfold thickness. These observations can be attributed to marker compounds (i.e., furostanol glycosides) of Fenu-FG supplementation. This furostanol glycoside (i.e., diosgenin) plays a role in hormone-interceded activity pathways. The glycoside fraction of fenugreek also demonstrated fat-burning and anabolic activity in animals [27].

Additionally, a previous study reported that Fenu-FG supplementation significantly increased the repetitions to failure (muscle endurance) during eight weeks of resistance training in males compared to the placebo group. However, Fenu-FG supplementation failed to increase muscle strength significantly [27]. Further, subjects who received fenugreek extracts (300 mg/kg) exhibited improved aerobic capacity after four weeks of treatment [34]. In a randomized, double-blind, placebo-controlled, parallel-design trial including 45 untrained women and men, the ingestion of FEN (300 mg/day) improved aerobic performance and delayed fatigue in healthy participants. However, the study did not cover “very poor” and “high” fitness individuals. It recommended that the variation between the curcumin and fenugreek supplementation on the ventilatory threshold can be revealed in future studies. Also, the effects of fenugreek fiber alone and curcumin with other ingredients (i.e., piperine) on submaximal endurance performance indices can be explored [23].

### 4.2. Ergogenic Aid

Athletes often seek natural formulations to enhance stamina, aerobic capacity, muscle growth, and overall performance [35]. Creatine monohydrate is widely investigated as a sport and consumable supplement. It is a predominant component of ergogenic aids and sports nutrition [24]. Moreover, it is an important energy source for muscles and is usually consumed by gymnasts and athletes during high-intensity, short-duration regimens [36]. It is phosphorylated into energy-generating substrates (e.g., phosphocreatine), replenishing adenosine triphosphate in active skeletal muscle [36].

Nearly half of the creatine is produced endogenously from amino acids; the remainder is obtained via supplementation or dietary protein consumption [37]. At the molecular level, in situ research demonstrated that fenugreek extracts resulted in greater insulin-stimulated intracellular creatine content in mock-transfected L6 (clone L6C11) skeletal myoblasts [38], which may offer an extra advantage for exercise performance. 

In addition, several studies stated that consuming carbohydrates and creatine simultaneously improves body composition and muscle strength [39,40]. Meanwhile, chronic consumption of high amounts of carbohydrates provides large amounts of calories and can have harmful health outcomes, such as changes in body composition and hyperglycemia [41]. A clinical trial using a structured resistance training program found that combining 3.5 g of creatine with 900 mg of a fenugreek extract for eight weeks had the same effect on male body composition and strength as combining 5 g creatine with 70 g dextrose, which indicates that fenugreek extracts have increased muscle creatine uptake without requiring excessive amounts of creatine or carbohydrates. Such alternative creatine supplementation might be helpful for those with the adverse effects of consumption of vast amounts of simple carbohydrates. In the future, fenugreek plus conventional creatine supplements can be analyzed to evaluate the ability of fenugreek to deliver and retain creatine within skeletal muscle compared to creatine-carbohydrate supplements. Further research can be conducted to ascertain the effect of fenugreek extract on androgen levels since alterations in hormone levels could influence resistance training adaptions [24].

In light of the established role of testosterone in androgen-regulating physiological activity and improving muscle strength and energy, marketed formulations focus on testosterone enhancer products [42]. Due to the unfavorable side effects of anabolic steroids, pharmaceutical companies are now trying to produce natural nutritional products containing ergogenic components. Importantly, fenugreek extracts have previously been shown to increase free testosterone hormone levels in older men with androgen decline and reduce physical symptoms associated with hypogonadism [43]. Similarly, 600 mg fenugreek extracts increased testosterone hormone levels from the baseline value [32].

Moreover, during an eight-week resistance training program, Fenu-FG supplementation demonstrated substantial anabolic and androgenic activity in males. It enhanced the serum-free testosterone without reduction in total testosterone [27]. The androgenic activity is supposed to have interceded through the 5-alpha-reductase and aromatase inhibition pathways. Also, the glycoside-rich fractions of fenugreek seeds, such as sapogenins and saponins, have shown anabolic and androgenic activities [27]. Notably, saponins, the compound protodioscin, influence the anabolic and testosterone status [44]. Furthermore, Fenu-FG supplementation significantly reduced serum creatinine and blood urea nitrogen levels, which are likely pointers of anabolic activity [27].

### 4.3. Post-Exercise Recovery

During physical activity, liver and muscle glycogen stores are the primary energy source, making them vital for success. When the muscle glycogen level is depleted, it must be resynthesized during recovery to achieve the highest level during subsequent exercise bouts [26]. Optimal post-exercise glycogen store repletion is a critical factor in achieving optimal performance, especially in athletes who train multiple times per day [45]. An earlier trial has shown that a fenugreek extract supplement with dextrose can promote and accelerate the glycogen resynthesis rate after the glycogen-depleting cycle exercise test [26]. However, another trial could not prove the positive effect of fenugreek extract on the muscle glycogen resynthesis rate [25]. Those trials were confined to minimal sample size and female subjects [25,26]. Further research is required to recognize fenugreek interactions in normoglycemic exercise in humans, and the conditions must provoke physiological effects [25]. Research is also required into the basic mechanisms of the unique amino acid (fenugreek seeds) and its effect on muscle glycogen recovery and subsequent exercise output during multiple days of laborious exercise if glycogen concentrations are reliably confronted [26]. These points might give rise to new research questions for future studies.

Additionally, ingesting amino acids or protein with carbohydrates in post-exercise sessions has been proposed to promote the glycogen synthase pathway [46,47]. Macronutrients, especially proteins, are associated with nutritional support to enhance recovery and return to competition and training after exercise-induced injuries and those involving immobilization [48]. Inadequate protein consumption can lead to health problems, impair wound healing, and likely increase inflammation and cytokines to a harmful level [49]. The reduction in protein (myofibrillar) synthesis affects muscle metabolism and results in muscle loss. Accordingly, the healing process mainly depends on collagen and other protein syntheses [50].

On the other hand, fenugreek containing 4-hydroxyisoclucine (nonproteinergic amino acid), apigenins, saponins, and alkaloids has notable anti-inflammatory activity [3]. This effect could help treat exercise-induced injuries due to the anti-inflammatory properties. Besides, the growth of fenugreek products intends to enhance their therapeutic efficacy through implementing techniques like nano-formulation and liposomal drug delivery. Nevertheless, future studies must reveal how fenugreek products inhibit inflammatory signals [3].

### 4.4. Health Benefits of Fenugreek

The health benefits of fenugreek seeds are feasible since those seeds contain fenugreek glycosides, namely, sapogenins (e.g., diosgenin), saponins, savsalpogenin, yuccagenin, and similagenin [27]. Among those glycosides, sapogenins and saponins have demonstrated anabolic and androgenic activities [27]. Moreover, an increase in serum testosterone level might be because fenugreek inhibits the enzymes like aromatase and 5-alpha-reductase and limits the metabolism of serum testosterone [27]. Diosgenin, a glycoside constituent of fenugreek, influences cholesterol metabolism. The fenugreek saponins do not interact directly with cholesterol; however, they have a robust inhibitory effect on bile salt absorption [27]. Fenugreek intake has elevated creatine absorption and retention, which results in earlier training adaptations. Also, it might influx creatine into the skeletal muscle like carbohydrates [24].

**Figure 4:**
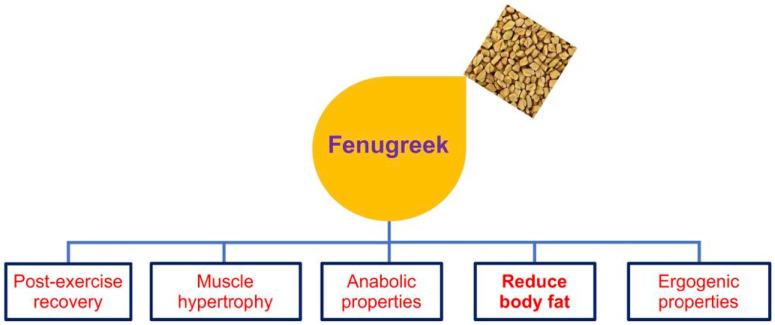
Diagram showing the multiple health benefits of fenugreek.

Based on the studies analyzed for review, it is suggested that fenugreek extracts are a natural plant supplement that positively affects muscle performance and recovery. The hypothesized mechanisms for enhancing muscle performance include increased anabolic and androgenic activity and rate of glycogen resynthesis following glycogen depletion. In line with these statements, fenugreek is a rich source of several nitrogen-containing amino acids, carbohydrates, and other bionutrient compounds, which improve exercise performance and muscle function recovery and delay fatigue (Figure 4). Additional studies are required to attain the potential doses and effects of fenugreek extracts as an efficacious nutraceutical or over-the-counter sports supplement.

Notably, all performance-improving supplements are regulated by the world anti-doping code as demarcated by the word anti-doping agency. Several conventional herbal medicines are being examined as safer substitutes for their nutritional aids and performance improvement. However, their safety and effectiveness must be scientifically investigated [27]. Though natural supplements in markets improve health and physical performance, it is sensible to be aware that some herbs might possess doping substances in their composition, and some products of herbal extracts might be adulterated by agents prohibited in sports activities [35]. Fenugreek supplementation is beneficial during resistance training without any clinical side effects; however, doping aspects should also be confirmed before using it for sports events. At the same time, Wankhede et al. showed that fenugreek supplementation positively affects free testosterone levels, serum creatinine, and body fat without causing changes in kidney profile enzymes [27]. However, more protein or amino acid intake might lead to severe liver damage and metabolic diseases [51]. It is advised to be cautious while consuming fenugreek as a sports supplement rich in nitrogen-containing amino acids, to prevent liver and kidney damage. Hence, further investigations are warranted to reveal the effect of fenugreek intake on liver and kidney functions. Also, it is noteworthy that some individuals might experience allergic reactions to fenugreek despite its safety and usefulness. Those allergic reactions include dyspepsia, abdominal distention, diarrhea, flatulence, and hypoglycemia [35].

Previous literature has intensively investigated stress-related disorders and chronic pain conditions with various objectives [52,53,54,55,56]. However, some studies focused on revealing fenugreek’s role in managing pain and stress-related conditions in animals and humans [57,58,59,60,61]. Future studies can explore the effect of fenugreek in alleviating pain and mental stress, especially among athletes. Moreover, a recent study revealed the antihyperglycemic effect of fenugreek and ginger in type 2 diabetes mellitus (T2DM) cases. It is reported that the oral intake of 1 g of fenugreek capsules three times a day for eight weeks significantly reduced fasting blood sugar and glycosylated hemoglobin compared to ginger capsules. Both fenugreek and ginger capsules significantly reduced serum creatinine and triglyceride levels in T2DM cases [62]. The precise mechanism behind these observations is still being determined. However, the possible hypoglycemic and lipid-reducing mechanism of fenugreek comprise late gastric emptying, inhibition of glucose transport, raised oxidative stress, low fat, and glucose absorption, enhanced modulation of glucagon-like peptide-1, augmented insulin sensitivity, and stimulation of insulin secretion [63]. Furthermore, combining fenugreek seed extracts and swimming training significantly decreased plasma glucose and enhanced cardiac antioxidant enzyme activity. However, those changes were observed in diabetic rats, not humans [64].

## 5. Conclusions

This review reported that fenugreek extracts positively reduced body fat, increased lean body mass, improved muscle strength and endurance, and accelerated the rate of glycogen resynthesis during post-exercise recovery. Their anabolic and androgenic activity is responsible for enhanced muscle performance. Further, those extracts act as an ergogenic aid and increase muscle creatine uptake to provide energy for the muscles during sports activities. However, the effects of various fenugreek extractions and doses on anabolic hormones influencing additional strength and power output adaptations can be discovered in future studies. Also, the impact of fenugreek fiber alone, and curcumin combined with piperine, on submaximal endurance performance indices can be revealed. Further research is warranted to cover a larger sample size while revealing the role of fenugreek extracts on the rate of glycogen resynthesis. Most included RCTs covered only male subjects, which insists the researchers should include both genders in the future. Though fenugreek supplementation is valuable during resistance training and deprived of clinical side effects, doping should be considered before using it for sports activities. It is necessary to ensure awareness of protecting liver and kidney function while consuming fenugreek as a sports supplement.

## Figures and Tables

**Table 1:** Summary of the included studies.

Studies	Location	Mean Age	Sample Size	Sex	Design	Daily Dose	Intervention/Control	Duration	Limitations	Quality Score
Wankhede et al. [27]	India	23 ± 3	60	M	DBRPC	300 mg/day	GF/PB	Eight weeks	Involved male participants only	6
Poole et al. [4]	USA	21 ± 2	49	M	DBRPC	500 mg	FG/PB	Eight weeks	Involved male participants only	6
Taylor et al. [24]	USA	21 ± 2	47	M	RPC	900 mg	FGE/PB	Eight weeks	Involved male participants only	5
Goh et al. [23]	USA	20 ± 1	45	M/F	DBRPC	300 mg/day	SF/PB	28 days	(i) Excluded the participants who were below fitness strata, i.e., “very poor” and “high” fitness. Hence, the findings cannot be generalized to individuals falling within those fitness categories, (ii) Dependence on subject compliance, and (iii) Participants are confined to the laboratory during the supplementation and testing periods; hence sleep and dietary intake outside of the three days prior to pre-and post-test were not accounted.	4
Ruby et al. [26]	USA	26 ± 5	6	M	DBRPC	2.0 mg.kg^−1^	FGE/PB	2 h	(i) Very small sample size (ii) Involved male participants only	3
Slivka et al. [25]	USA	28 ± 9	8	M	DBRPC	1.99 ± 0.20 mg.kg^−1^	FGE/PB	15 h	(i) Very small sample size (ii) Involved male participants only	3

M, male; F, female; FGE, fenugreek seed extract; GF, glycoside fraction of fenugreek seeds; PB, placebo; SF, fenugreek soluble fiber; DBRPC, double-blind, randomized, placebo-controlled study; RPC, randomized, placebo-controlled design; FG, fenugreek.

## Data Availability

Not applicable.

## References

[1] Mazza G., Di Tommaso D., Foti S. (2002). Volatile constituents of Sicilian fenugreek (*Trigonella foenum-graecum* L.) seeds. Sci. Aliment..

[2] Shashikumar J.N., Champawat P.S., Mudgal V.D., Jain S.K., Deepak S., Mahesh K. (2018). A review: Food, medicinal and nutraceutical properties of fenugreek (*Trigonella foenum-graecum* L.). Int. J. Chem. Stud..

[3] Nagulapalli Venkata K.C., Swaroop A., Bagchi D., Bishayee A. (2017). A small plant with big benefits: Fenugreek (*Trigonella foenum-graecum* Linn.) for disease prevention and health promotion. Mol. Nutr. Food Res..

[4] Poole C., Bushey B., Foster C., Campbell B., Willoughby D., Kreider R., Taylor L., Wilborn C. (2010). The effects of a commercially available botanical supplement on strength, body composition, power output, and hormonal profiles in resistance-trained males. J. Int. Soc. Sports Nutr..

[5] Haghani K., Bakhtiyari S., Doost Mohammadpour J. (2016). Alterations in plasma glucose and cardiac antioxidant enzymes activity in streptozotocin-induced diabetic rats: Effects of *Trigonella foenum-graecum* extract and swimming training. Can. J. Diabetes.

[6] Sheweita S.A., ElHady S.A., Hammoda H.M. (2020). Trigonella stellata reduced the deleterious effects of diabetes mellitus through alleviation of oxidative stress, antioxidant- and drug-metabolizing enzymes activities. J. Ethnopharmacol..

[7] Mahapatra K., Ghosh A.K., De S., Ghosh N., Sadhukhan P., Chatterjee S. (2020). Assessment of cytotoxic and genotoxic potentials of a mononuclear Fe(II) Schiff base complex with photocatalytic activity in Trigonella. Biochim. Biophys. Acta Gen. Subj..

[8] Afroz R., Rahman K.A., Lotus M.J., Afrin T., Yeasmin N., Moon K.J. (2020). Histopathological evaluation of gastro protective effect of *Trigonella foenum Graecum* seed (Methi) and omeprazole in experimentally induced gastric ulcer in rats. J. Dhaka Med. College.

[9] Nagamma T., Konuri A., Bhat K.M.R., Maheshwari R., Udupa P., Nayak Y. (2021). Modulation of inflammatory markers by petroleum ether fraction of *Trigonella foenum-graecum* L. seed extract in ovariectomized rats. J. Food Biochem..

[10] Al-Timimi L.A.N. (2019). Antibacterial and anticancer activities of fenugreek seed extract. Asian Pac. J. Cancer Prev..

[11] Moustafa E.M., Dawood M.A., Assar D.H., Omar A.A., Elbialy Z.I., Farrag F.A., Shukry M., Zayed M.M. (2020). Modulatory effects of fenugreek seeds powder on the histopathology, oxidative status, and immune related gene expression in Nile tilapia (*Oreochromis niloticus*) infected with *Aeromonas hydrophila*. Aquaculture.

[12] Gao F., Du W., Zafar M.I., Shafqat R.A., Jian L., Cai Q., Lu F. (2015). 4-Hydroxyisoleucine ameliorates an insulin resistant-like state in 3T3-L1 adipocytes by regulating TACE/TIMP3 expression. Drug Des. Devel. Ther..

[13] Belaïd-Nouira Y., Bakhta H., Haouas Z., Flehi-Slim I., Neffati F., Najjar M.F., Cheikh H.B. (2013). Fenugreek seeds, a hepatoprotector forage crop against chronic AlCl3 toxicity. BMC Vet. Res..

[14] Doshi M., Mirza A., Umarji B., Karambelkar R. (2012). Effect of *Trigonella foenum-graecum* (fenugreek/methi) on hemoglobin levels in females of child bearing age. Biomed. Res..

[15] Zameer S., Najmi A.K., Vohora D., Akhtar M. (2018). A review on therapeutic potentials of *Trigonella foenum graecum* (fenugreek) and its chemical constituents in neurological disorders: Complementary roles to its hypolipidemic, hypoglycemic, and antioxidant potential. Nutr. Neurosci..

[16] Konuri A., Bhat K.M.R., Rai K.S., Gourishetti K., Phaneendra M.Y.S. (2021). Supplementation of fenugreek with choline–docosahexaenoic acid attenuates menopause induced memory loss, BDNF and dendritic arborization in ovariectomized rats. Anat. Sci. Int..

[17] Mansoori A., Hosseini S., Zilaee M., Hormoznejad R., Fathi M. (2020). Effect of fenugreek extract supplement on testosterone levels in male: A meta-analysis of clinical trials. Phytother. Res..

[18] Alcantara J.M.A., Sanchez-Delgado G., Martinez-Tellez B., Labayen I., Ruiz J.R. (2019). Impact of cow’s milk intake on exercise performance and recovery of muscle function: A systematic review. J. Int. Soc. Sports Nutr..

[19] McCubbin A.J., Allanson B.A., Odgers J.N., Cort M.M., Costa R.J., Cox G.R., Crawshay S.T., Desbrow B., Freney E.G., Gaskell S.K. (2020). Sports Dietitians Australia position statement: Nutrition for exercise in hot environments. Int. J. Sport Nutr. Exerc. Metab..

[20] Areta J.L., Burke L.M., Ross M.L., Camera D.M., West D.W., Broad E.M., Jeacocke N.A., Moore D.R., Stellingwerff T., Phillips S.M. (2013). Timing and distribution of protein ingestion during prolonged recovery from resistance exercise alters myofibrillar protein synthesis. J. Physiol..

[21] Page M.J., Moher D., Bossuyt P.M., Boutron I., Hoffmann T.C., Mulrow C.D., Shamseer L., Tetzlaff J.M., Akl E.A., Brennan S.E. (2021). PRISMA 2020 explanation and elaboration: Updated guidance and exemplars for reporting systematic reviews. BMJ.

[22] Hayden J.A., Côté P., Bombardier C. (2006). Evaluation of the quality of prognosis studies in systematic reviews. Ann. Intern. Med..

[23] Goh J., Menke W., Herrick L.P., Campbell M.S., Abel M.G., Fleenor B.S., Bergstrom H.C. (2020). Examination of curcumin and fenugreek soluble fiber supplementation on submaximal and maximal aerobic performance indices. J. Funct. Morphol. Kinesiol..

[24] Taylor L., Poole C., Pena E., Lewing M., Kreider R., Foster C., Wilborn C. (2011). Effects of combined creatine plus fenugreek extract vs. creatine plus carbohydrate supplementation on resistance training adaptations. J. Sports Sci. Med..

[25] Slivka D., Cuddy J., Hailes W., Harger S., Ruby B. (2008). Glycogen resynthesis and exercise performance with the addition of fenugreek extract (4-hydroxyisoleucine) to post exercise carbohydrate feeding. Amino Acids.

[26] Ruby B.C., Gaskill S.E., Slivka D., Harger S.G. (2005). The addition of fenugreek extract (*Trigonella foenum-graecum*) to glucose feeding increases muscle glycogen resynthesis after exercise. Amino Acids.

[27] Wankhede S., Mohan V., Thakurdesai P. (2016). Beneficial effects of fenugreek glycoside supplementation in male subjects during resistance training: A randomized controlled pilot study. J. Sport Health Sci..

[28] Aydogan U., Eroglu A., Akbulut H., Yildiz Y., Gok D.E., Sonmez A., Aydin T., Bolu E., Saglam K. (2012). Evaluation of the isokinetic muscle strength, balance and anaerobic performance in patients with young male hypogonadism. Endocr. J..

[29] Basualto-Alarcón C., Jorquera G., Altamirano F., Jaimovich E., Estrada M. (2013). Testosterone signals through mTOR and androgen receptor to induce muscle hypertrophy. Med. Sci. Sports Exerc..

[30] Bajer B., Vlcek M., Galusova A., Imrich R., Penesova A. (2015). Exercise associated hormonal signals as powerful determinants of an effective fat mass loss. Endocr. Regul..

[31] Aswar U., Mohan V., Bhaskaran S., Bodhankar S.L. (2008). Study of galactomanan on androgenic and anabolic activity in male rats. Pharmacol. Online Young Res..

[32] Rao A.J., Mallard A.R., Grant R. (2020). Testofen^®^ (Fenugreek extract) increases strength and muscle mass compared to placebo in response to calisthenics. A randomized control trial. Transl. Sports Med..

[33] Woodgate D.E., Conquer J.A. (2003). Effects of a stimulant-free dietary supplement on body weight and fat loss in obese adults: A six-week exploratory study. Curr. Ther. Res. Clin. Exp..

[34] Ikeuchi M., Yamaguchi K., Koyama T., Sono Y., Yazawa K. (2006). Effects of fenugreek seeds (Trigonella foenum greaecum) extract on endurance capacity in mice. J. Nutr. Sci. Vitaminol..

[35] Sellami M., Slimeni O., Pokrywka A., Kuvačić G., DHayes L., Milic M., Padulo J. (2018). Herbal medicine for sports: A review. J. Int. Soc. Sports Nutr..

[36] Greenhaff P.L. (2001). The creatine-phosphocreatine system: There’s more than one song in its repertoire. J. Physiol..

[37] Jäger R., Purpura M., Shao A., Inoue T., Kreider R.B. (2011). Analysis of the efficacy, safety, and regulatory status of novel forms of creatine. Amino Acids.

[38] Tomcik K.A., Smiles W.J., Camera D.M., Hügel H.M., Hawley J.A., Watts R. (2017). Fenugreek increases insulin-stimulated creatine content in L6C11 muscle myotubes. Eur. J. Nutr..

[39] Becque M.D., Lochmann J.D., Melrose D.R. (2000). Effects of oral creatine supplementation on muscular strength and body composition. Med. Sci. Sports Exerc..

[40] Bemben M.G., Bemben D.A., Loftiss D.D., Knehans A.W. (2001). Creatine supplementation during resistance training in college football athletes. Med. Sci. Sports Exerc..

[41] Albaker W., El-Ashker S., Baraka M.A., El-Tanahi N., Ahsan M., Al-Hariri M. (2021). Adiposity and cardiometabolic risk assessment among university students in Saudi Arabia. Sci. Prog..

[42] Matsumoto A.M. (2002). Andropause: Clinical implications of the decline in serum testosterone levels with aging in men. J. Gerontol. A Biol. Sci. Med. Sci..

[43] Rao A., Steels E., Inder W.J., Abraham S., Vitetta L. (2016). Testofen, a specialised *Trigonella foenum-graecum* seed extract reduces age-related symptoms of androgen decrease, increases testosterone levels and improves sexual function in healthy aging males in a double-blind randomised clinical study. Aging Male.

[44] Pavin N.F., Izaguirry A.P., Soares M.B., Spiazzi C.C., Mendez A.S.L., Leivas F.G., Brum D.S., Cibin F.W.S. (2018). *Tribulus terrestris* protects against male reproductive damage induced by cyclophosphamide in mice. Oxid. Med. Cell Longev..

[45] Craven J., Desbrow B., Sabapathy S., Bellinger P., McCartney D., Irwin C. (2021). The effect of consuming carbohydrate with and without protein on the rate of muscle glycogen re-synthesis during short-term post-exercise recovery: A systematic review and meta-analysis. Sports Med. Open.

[46] Ivy J.L., Lee M.C., Brozinick J.T., Reed M.J. (1988). Muscle glycogen storage after different amounts of carbohydrate ingestion. J. Appl. Physiol..

[47] Zawadzki K.M., Yaspelkis B.B., Ivy J.L. (1992). Carbohydrate-protein complex increases the rate of muscle glycogen storage after exercise. J. Appl. Physiol..

[48] Tipton K.D. (2015). Nutritional support for exercise-induced injuries. Sports Med..

[49] Demling R.H. (2009). Nutrition, anabolism, and the wound healing process: An overview. Eplasty.

[50] Lorenz H.P., Longaker M.T., Norton J.A., Barie P.S., Bollinger R.R., Chang A.E., Lowry S.F., Mulvihill S.J., Pass H.I., Tompson R.W. (2008). Wounds: Biology, Pathology, and Management. Surgery: Basic Science and Clinical Evidence.

[51] Lee D.Y., Kim E.H. (2019). Therapeutic Effects of Amino Acids in Liver Diseases: Current Studies and Future Perspectives. J. Cancer Prev..

[52] Alvarez-Mon M.A., Gómez-Lahoz A.M., Orozco A., Lahera G., Diaz D., Ortega M.A., Albillos A., Quintero J., Aubá E., Monserrat J. (2021). Expansion of CD4 T Lymphocytes Expressing Interleukin 17 and Tumor Necrosis Factor in Patients with Major Depressive Disorder. J. Pers. Med..

[53] Bremner J.D., Gurel N.Z., Wittbrodt M.T., Shandhi M.H., Rapaport M.H., Nye J.A., Pearce B.D., Vaccarino V., Shah A.J., Park J. (2020). Application of Noninvasive Vagal Nerve Stimulation to Stress-Related Psychiatric Disorders. J. Pers. Med..

[54] Di Lernia D., Lacerenza M., Ainley V., Riva G. (2020). Altered Interoceptive Perception and the Effects of Interoceptive Analgesia in Musculoskeletal, Primary, and Neuropathic Chronic Pain Conditions. J. Pers. Med..

[55] Guerrini Usubini A., Cattivelli R., Varallo G., Castelnuovo G., Molinari E., Giusti E.M., Pietrabissa G., Manari T., Filosa M., Franceschini C. (2021). The Relationship between Psychological Distress during the SecondWave Lockdown of COVID-19 and Emotional Eating in Italian Young Adults: The Mediating Role of Emotional Dysregulation. J. Pers. Med..

[56] Halaris A., Sohl E., Whitham E.A. (2021). Treatment-Resistant Depression Revisited: A Glimmer of Hope. J. Pers. Med..

[57] Hausenblas H.A., Conway K.L., Coyle KR M., Barton E., Smith L.D., Esposito M., Harvey C., Oakes D., Hooper D.R. (2020). Efficacy of fenugreek seed extract on men’s psychological and physical health: A randomized placebo-controlled double-blind clinical trial. J. Complement. Integr. Med..

[58] Kooshki A., Khazaei Z., Rad M., Zarghi A., Mogaddam A. (2018). Effects of fenugreek seed powder on stress-induced hyperglycemia and clinical outcomes in critically ill patients: A randomized clinical trial. Biomed. Res. Ther..

[59] Pandaran Sudheeran S., Jacob D., Natinga Mulakal J., Gopinathan Nair G., Maliakel A., Maliakel B., Kuttan R., Im K. (2016). Safety, Tolerance, and Enhanced Efficacy of a Bioavailable Formulation of Curcumin With Fenugreek Dietary Fiber on Occupational Stress: A Randomized, Double-Blind, Placebo-Controlled Pilot Study. J. Clin. Psychopharmacol..

[60] Sindhu G., Shyni G.L., Pushpan C.K., Bala Nambisan B., Helen A. (2018). (2018). Evaluation of anti-arthritic potential of *Trigonella foenum graecum* L. (Fenugreek) mucilage against rheumatoid arthritis. Prostaglandins Other Lipid Mediat..

[61] Younesy S., Amiraliakbari S., Esmaeili S., Alavimajd H., Nouraei S. (2014). Effects of fenugreek seed on the severity and systemic symptoms of dysmenorrhea. J. Reprod. Infertil..

[62] Elsaadany M.A., AlTwejry H.M., Zabran R.A., AlShuraim S.A., AlShaia W.A., Abuzaid O.I., AlBaker W.I. (2022). Antihyperglycemic Effect of Fenugreek and Ginger in Patients with Type 2 Diabetes: A Double-Blind, Placebo-controlled Study. Curr. Nutr. Food Sci..

[63] Fuller S., Stephens J.M. (2015). Diosgenin, 4-hydroxyisoleucine, and fiber from fenugreek: Mechanisms of actions and potential effects on metabolic syndrome. Adv. Nutr..

[64] Arshadi S., Bakhtiyari S., Bakhtiyari S., Haghani K., Valizadeh A. (2015). Effects of Fenugreek Seed Extract and Swimming Endurance Training on Plasma Glucose and Cardiac Antioxidant Enzymes Activity in Streptozotocin-induced Diabetic Rats. Osong Public Health Res. Perspect..

